# Sneaky tactics: Ingenious immune evasion mechanisms of *Bartonella*

**DOI:** 10.1080/21505594.2024.2322961

**Published:** 2024-03-05

**Authors:** Yixuan Xi, Xinru Li, Lu Liu, Feichen Xiu, Xinchao Yi, Hongliang Chen, Xiaoxing You

**Affiliations:** a Institute of Pathogenic Biology, Hunan Provincial Key Laboratory for Special Pathogens Prevention and Control, Hunan Province Cooperative Innovation Center for Molecular Target New Drug Study, Hengyang Medical College, University of South China, Hengyang, China; b Chenzhou NO.1 People’s Hospital, The Affiliated Chenzhou Hospital, Hengyang Medical College, University of South China, ChenZhou, China

**Keywords:** *Bartonella*, antigen variation, intracellular survival, biofilm, apoptosis

## Abstract

Gram-negative *Bartonella* species are facultative intracellular bacteria that can survive in the harsh intracellular milieu of host cells. They have evolved strategies to evade detection and degradation by the host immune system, which ensures their proliferation in the host. Following infection, *Bartonella* alters the initial immunogenic surface-exposed proteins to evade immune recognition via antigen or phase variation. The diverse lipopolysaccharide structures of certain *Bartonella* species allow them to escape recognition by the host pattern recognition receptors. Additionally, the survival of mature erythrocytes and their resistance to lysosomal fusion further complicate the immune clearance of this species. Certain *Bartonella* species also evade immune attacks by producing biofilms and anti-inflammatory cytokines and decreasing endothelial cell apoptosis. Overall, these factors create a challenging landscape for the host immune system to rapidly and effectively eradicate the *Bartonella* species, thereby facilitating the persistence of *Bartonella* infections and creating a substantial obstacle for therapeutic interventions. This review focuses on the effects of three human-specific *Bartonella* species, particularly their mechanisms of host invasion and immune escape, to gain new perspectives in the development of effective diagnostic tools, prophylactic measures, and treatment options for *Bartonella* infections.

## Introduction

*Bartonella* species are gram-negative, facultative, and zoonotic bacteria belonging to the α2-subgroup of proteobacteria. They exhibit distinct metabolic traits and are negative for catalase, oxidase, urease, and nitrate reductase tests [1]. These pathogens primarily target mammals and rely on blood-sucking arthropod vectors for transmission. To date, approximately 20 *Bartonella* species have been identified as the causative agents of host-specific intraerythrocytic infections in mammals. Notable among them are the human-specific pathogens *Bartonella henselae*, *B. quintana*, and *B. bacilliformis*. Generally, *B. henselae* and *B. quintana* cause limited morbidity, often manifesting as the cat scratch disease (CSD) or trench fever. In contrast, *B. bacilliformis* is a lethal pathogen that leads to Carrion’s disease, resulting in the death of approximately 80% of the affected individuals in the acute stage; however, this is limited to the Andes area [2–5]. It is probably that *Bartonella* species are geographically widespread because of the regional specificities of their respective mammalian hosts and arthropod vectors [6,7], For instance, the seroprevalence of *Bartonella* is high in the European Mediterranean countries, where flea and tick infestations are favoured by high temperatures and humidity [8]. CSD leads to approximately 12, 000 outpatient diagnoses and 500 hospitalizations annually in the United States. The highest incidence of CSD is observed in southern United States (0.0064%), especially among children aged 5–9 years (0.0094%) [9]. Nonetheless, the incidence of trench fever caused by *B. quintana* has dropped considerably since World War I. Currently, it is primarily associated with homelessness, alcoholism, destitution, and unfavourable living conditions, particularly in terms of health and hygiene, within the marginalized population of developed nations [10–12].

Infection caused by *Bartonella* is an overly complex process that involves multiple virulence factors and biological phenomena. Apart from *B. bacilliformis*, *B. henselae* and *B. quintana* use their common VirB/D4-type 4 secretion system (T4SS) and various *Bartonella* effector proteins (Beps) as major virulence factors to hinder host cell apoptosis, enabling bacterial persistence in erythrocytes and promoting endothelial sprouting. However, pathogens with unique flagellar structures, such as *B. bacilliformis* and *B. clarridgeiae* have unique flagellar structures, they usually utilize the motility of the flagella and membrane-associated protein, invasion-associated locus A/B (IalA/B), to enter the mature human erythrocytes [13]. After transmission, the pathogen settles in a primary location that is as of yet unresolved, such as the migrating cells, vascular endothelium, lymph nodes, mature erythrocytes, and even human stem cells, in order to complete its developmental life cycle [14–18].

Persistent bacteraemia within erythrocytes appears to be a specialized adjustment to the transmission of *Bartonella* in the host species [10,19,20]. Specifically, dendritic cells (DCs) within CSD granulomas in *Bartonella*-infected immune-competent humans can also restrict infection to the lymph node region by means of a localized humoral immune response facilitated by B cells [21]. In contrast, immunocompromised individuals exhibit a heightened vulnerability to bacteraemia, thereby amplifying the potential for remote microbial dissemination. Historically, clinical and *in vitro* studies have revealed that *B. henselae* infection triggers immune responses, including delayed-type hypersensitivity and release of interferon-γ, primarily via CD4^+^ Th1 cells in immune-competent mice [22,23]. Notwithstanding the presence of such robust immune countermeasures, effective immune retaliation against *Bartonella* remains an arduous task for the host, which contributes to the persistence and proliferation of this pathogen. Various studies have revealed the increase in the expression levels of cytokines, such as interleukin (IL)-2, IL-6, and IL-10, in patients with CSD [24]. Owing to the inherent anti-inflammatory attributes of IL-10, such cytokines disrupt the balance, leading to sustained bacterial infection [24–26].

**Figure 1.**
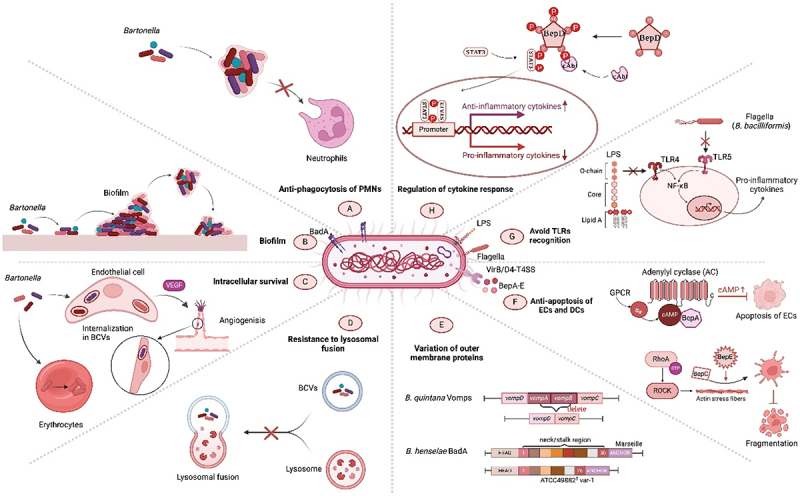
Overview of the immune evasion strategies of pathogenic *Bartonella* species. The robust cytoadhesive capacity of *Bartonella* adhesin a (BadA) helps to establish “invasomes,” thus evading phagocytosis by host polymorphonuclear neutrophils (PMNs) and promoting infection (A). Some strains, such as *B. henselae*, form protective biofilms, shielding them from harsh conditions (B). *Bartonella* species use intricate intracellular mechanisms, including induction of angiogenesis, to survive and replicate in the host cells (C). Sometimes after invasion, *Bartonella* produce *Bartonella*-containing vacuoles (BCVs), which lack typical endocytic markers and cannot fuse with lysosomes, to evade degradation (D). *Bartonella* species occasionally alter the host surface proteins to evade detection via antigenic or phase variation (E). Continuously growing *Bartonella* species also reduce endothelial cell susceptibility to apoptosis, mediated by the *Bartonella* effector proteins (beps), enhancing their persistence (F). Distinctive pathogen-associated molecular patterns (PAMPs), such as lipopolysaccharides (LPSs), and flagella affect the toll-like receptor (TLR) recognition, weakening the immune response (G). *Bartonella* species use various mechanisms to regulate anti-inflammatory cytokine secretion, reducing the efficacy of therapeutic strategies to disrupt pro/anti-inflammatory balance. These factors enable prolonged infection and pose significant challenges for effective treatment (H).

In hostile environments, *Bartonella* species encounter formidable challenges, particularly incessant surveillance and potential degradation by host immune system. Therefore, these bacteria have developed various sneaky tactics to elude the host immune response and ensure their survival within these cellular confines. These include resistance to immune clearance by escaping phagocytic engulfment, survival of erythrocytes and endothelial cells (ECs), and resistance to lysosomal fusion. Additionally, *Bartonella* alters the surface-exposed proteins and diverse lipopolysaccharide (LPS) structures that are not easily detected by the host immune system. Survival of *Bartonella* within the host is facilitated by its insensitivity to apoptosis, reduced toll-like receptor (TLR) recognition, and anti-inflammatory cytokines (Figure 1). Although not all *Bartonella* species exhibit similar pathogenic and immune evasion mechanisms, a comprehensive understanding of these intricate evasion tactics is paramount, offering invaluable insights that can be harnessed for the enhancement of diagnostic modalities, prophylactic interventions, and therapeutic regimens targeting *Bartonella* infections.

## Escape from phagocytic engulfment

The pivotal role of adhesins in facilitating the colonization and infiltration of host cells by pathogenic microorganisms is widely acknowledged in the field. However, some adhesins have been found to exhibit anti-phagocytic effects, thereby reducing the phagocytosis of polymorphonuclear neutrophils (PMNs) [27]. *Yersinia enterocolitica* adhesin A exerts anti-phagocytic effects to skilfully evade phagocytosis by PMNs. *Bartonella* adhesin A (BadA), an adhesin expressed by *B. henselae*, exhibits similar anti-phagocytic effects, as demonstrated by its ability to evade phagocytosis by J774 macrophages [28]. However, the exact mechanisms underlying the anti-phagocytic effects of BadA remain unclear, and no other *Bartonella* spp. with similar characteristics have been reported to date. It is possible that the biofilm produced by BadA may contribute to impaired phagocytosis by PMNs, as large bacterial aggregates are less likely to be engulfed by PMNs [29]. Therefore, it can be speculated that the strong cytoadhesive ability of BadA possibly enables bacteria to form relatively large “invasomes,” which facilitate the evasion of phagocytosis by PMNs and promote *Bartonella* infections in reservoir hosts.

## Biofilms

Biofilms are structured bacterial assemblies that adhere to biotic or abiotic surfaces and are encapsulated in a matrix of bacterial extracellular macromolecules. This matrix is enriched with an array of key biomolecules, including proteins, polysaccharides, DNA, RNA, peptidoglycans, lipids, and phospholipids [30]. Owing to the physical barrier provided by the biofilm matrix and its unique microenvironment, biofilms not only bolster the metabolic activities of resident bacteria but also facilitate inter-bacterial signalling [31]. Biofilms harbour essential enzymes and nutrients, facilitate message transfer, recycle components from lysed cells, e bacterial resilience in the host [32], all of which facilitate their long-term survival. Critically, biofilm architecture augments bacterial resistance against external threats, mainly antibiotics, and immune defence responses, including macrophage phagocytosis. This resilience often culminates in recurrent bacteraemia and persistent chronic infection in the host. In early 2003, Kyme et al. first reported that *B. henselae* has the ability to produce biofilms *in vitro*, similar to other bacteria; however, such biofilm-forming proficiency has not been reported in other *Bartonella* species yet [33].

Biofilm formed by *B. henselae* Houston-1 exhibits morphological characteristics similar to those of other bacterial biofilms; however, it exhibits distinct susceptibility to enzymatic degradation. Enzymatic actions of proteinase K and DNase impede biofilm formation in *B. henselae*. Environmental factors, including pH and temperature, play pivotal roles in modulating biofilm formation [34]. Okaro et al. reported that the optimal temperature for biofilm formation is 27 ℃. Moreover, biofilm formation gradually decreases with the increase in temperature, which partially explains the persistent survival of *Bartonella* in arthropods, even though the optimal temperature range for arthropod growth is generally 20–30 ℃. In neutral or weakly acidic environments, *B. henselae* exhibits enhanced propensity for biofilm formation [34].

Biofilm formation is an intricate process requiring the involvement of various virulence factors. Adhesion or aggregation is the first step for bacteria to form a biofilm [35], and this process is closely related to the trimeric autotransporter adhesin (TAA), BadA, because the expression level of BadA is parallel to the formation of biofilms at different temperatures [34,36]. This correlation underscores the notion that BadA-mediated aggregation may serve as a linchpin in biofilm formation. Corroborating this, recent evidence suggests that in various strains of *B. henselae*, the deletion of *badA* results in the appearance of a biofilm-negative phenotype [37,38]. However, a small amount of biofilm could still be formed by *badA* mutants [34], suggesting that other adhesins of *B. henselae* might also be involved in biofilm formation.

Biofilm genesis is a pivotal juncture in the lifecycle of *Bartonella*. Notably, *B. henselae* is postulated to orchestrate biofilm development within the alimentary milieu of its arthropod vectors, such as cat fleas, thus fortifying its ability to endure and proliferate. In the clinical setting, are the biofilms of *B. henselae*, sporadically identified as constituents of heart valve vegetation in patients with contracted blood culture-negative endocarditis. According to Okaro et al., an astounding eight out of 50 *Bartonella* species have been linked to infectious endocarditis [37]. Due to the potentially lower sensitivity of biofilms to antibiotics compared to free bacteria, *Bartonella* endocarditis cases with negative cultures may easily result in clinical misdiagnosis. Moreover, investigations at the molecular and histopathological levels of heart valves in individuals with *Bartonella* endocarditis have revealed a significant tendency for biofilm accumulation on the affected valve surfaces. Such compromised cardiac structures appear to provide a nurturing niche, fostering the inception and perpetuation of *Bartonella* biofilms, thereby leading to significant difficulties in the clinical treatment of bacterial infections [37]. Therefore, a nuanced understanding of the intricacies underlying *Bartonella* biofilm development and its attributes is imperative to devise efficacious therapeutic interventions.

Overall, the ability of *Bartonella* to create durable biofilms plays a role in its ability to persist in hosts. Based on the characteristics of biofilms, it is not challenging to envision that they represent an additional environment as a platform for introducing planktonic cells into the bloodstream, resulting in immune responses from the host that endure despite antimicrobial therapies. Nonetheless, the role of *Bartonella* biofilms in pathogenesis remains unclear, particularly concerning their prowess in thwarting phagocytosis and withstanding assault from host phagocytes. For example, to gain access to the bacteria residing in biofilms, neutrophils must initially fragment the biofilms into smaller fragments. Although the stress required for neutrophils to break apart from the biofilm is seemingly comparable to the elastic modulus of the biofilm, neutrophils are considerably smaller than the biofilm [39], thus it can be speculated that the biomechanics of biofilms might serve as formidable impediments, stymieing neutrophil-mediated disruption of biofilm-forming *Bartonella* species [40]. Hence, more in-depth studies on the capability of *B. henselae* biofilms to counteract host phagocytosis and evade immune responses are needed.

## Invasion of host cells

The intricate infection cycle of *Bartonella* commences with its introduction into mammalian hosts, which is predominantly mediated by haematophagous arthropod vectors. Notably, although erythrocytes constitute the principal target of invasion, *Bartonella* does not directly assail mature erythrocytes immediately after inoculation. In contrast, it persists in the primary niche before establishing a blood-stage infection [41]. Additionally, some studies have suggested that lymph nodes act as vehicles for *Bartonella* transport within this primary niche [19]. In subsequent phases, *Bartonella* disseminates throughout the blood vessels and invades mature erythrocytes, following a series of processes involving adhesion, eventual invasion, and persistence, thus facilitating sustained transmission of the vectors. Although variations may exist across distinct species, the basic idea of this infection cycle is typically preserved among *Bartonella spp*..

It has been identified that a variety of cell types serve as targets for *Bartonella* invasion, including ECs [42], endothelial progenitor cells [43], epithelial cells [44], haematopoietic progenitor cells (HPCs) [17], and monocytes or macrophages [45,46], serve as targets for invasion in *B. henselae*. Additionally, *B. bacilliformis* has been found to invade human dermal fibroblasts, laryngeal epithelial cells, human umbilical vein ECs (HUVECs) [47] as well and HeLa cells. Such extensive cellular tropism is postulated to underlie the diverse clinical manifestations associated with *Bartonella* infections. Once *Bartonella* adheres to their host cells, it triggers bacterial engulfment and cellular uptake or internalization. The mechanism of bacterial internalization in nucleated cells differs significantly from that in erythrocytes, owing to the distinct cytoskeleton structure of these two cell types. The main virulence factors mediating the entry of human pathogenic *Bartonella* into nucleated and red blood cells are listed in Table 1.

**Table 1.** Virulence factors related to the penetration of *bartonella* species into the host nucleated cells and erythrocytes.

Invasive factor(s)	Species	Direct function(s)	Contribution(s) to invasion	References	
Trimer autotransporter adhesins (BadA and Vomps)	*B. henselae; B. quintana*	Bacterial autoaggregation and adhesion to ECM, angiogenesis	Stable interaction with host cells promotes the secretion of angiopoietin		[3,28,48]
VirB/D4-T4SS Beps	*B. henselae; B. quintana*	Suppression of apoptosis and invasome formation, modulation of angiogenesis	Establishment and control of the intracellular niche		[13,49,50]
Trw type IV secretion system	*B. henselae; B. quintana*	Adhesion to the erythrocyte surface	Enabling erythrocyte invasion (lineage 4)		[50–52]
Deformin	*B. bacilliformis*	Creation of invaginations in the erythrocyte membrane	Enabling erythrocyte invasion		[53]
Heme-binding proteins, Hbp/Pap 31	*B. henselae; B. quintana*	Candidate outer membrane proteins and survival	Host cell adhesion and nutrient storage		[54–56]
Pili	*B. henselae; B. quintana*	Twitching motility and cell adhesion	Binding to the host cells		[1]
Flagellation	*B. bacilliformis*	Motility	Invasion of erythrocytes (lineages 1–3)		[57–59]
OMP43	*B. henselae*	Putative adhesin for endothelial cells	Invasion of endothelial cells		[42,54]
Invasion-associated locus (IalAB)	*B. bacilliformis*	Candidate OMPs of *Bartonella*	Invasion of erythrocytes		[60–62]

### Entry of Bartonella into nucleated cells

The sophisticated interactions between *Bartonella* and nucleated host cells, notably ECs, have been sufficiently elucidated *in vitro* studies. *B. henselae* enters human ECs via endocytosis to produce *Bartonella*-containing vacuoles (BCVs) or via invasome-mediated internalization. In the early stage of infection, the invasion process is largely dependent on the outer membrane proteins (OMPs) of *Bartonella*, among which TAAs, for instance, BadA of *B. henselae* [28,63], variably expressed outer membrane proteins (Vomps) of *B. quintana* [64] and *Bartonella* repeat proteins of *B. bacilliformis* [65] which are crucial for attaching to various extracellular matrix (ECM) components (collagen, laminin, fibronectin [Fn]) and membrane proteins of host cells. Other OMPs of *B. henselae*, such as Omp43, Omp89, Pap31 [54], and Bad-like YP_033004 (homologue of BadA) [48], also exhibit the ability to bind to intact ECM. Among them, Omp43, Omp89, and Pap31 are considered the three major putative Fn-binding proteins [143]. After adhesion, individual bacteria or small clusters are internalized into BCVs and enriched in the perinuclear region. Intriguingly, these BCVs resist acidification and lysosomal fusion by accumulating in the perinuclear region [16,42,66]. Significantly, similar BCVs have been identified in *B. bacilliformis* [67] and *B. quintana* [68], suggesting a potentially universal invasion strategy among *Bartonella* species, although the former lacks the VirB/D4-T4SS. In contrast, the invasome-mediated internalization pathway is multifaceted. This initiates bacterial accumulation and aggregation on the cell exterior, leading to subsequent engulfment of the bacterial aggregate through protrusions of the host cell membrane, which ultimately results in thorough bacterial internalization. This intricate mechanism is to a great extent orchestrated by Beps; for example, BepG can interfere with bacterial uptake of BCVs and trigger invasome-mediated internalization [69,70], ultimately affecting bacterial aggregate transportation. Invasome-mediated internalization is contingent upon bidirectional signal transduction through integrin receptors [51], as well as extensive rearrangements of the F-actin cytoskeleton [42]. Key molecular players, such as the Rho GTPases Rac1 and Cdc42, along with their allied effectors (e.g. Scar/WAVE, WASP, and PAK-1) and the Arp2/3 complex, which is pivotal for actin nucleation, are indispensable for cytoskeletal modifications and subsequent invasome formation [69–71 and 144]. Furthermore, the tyrosine kinases Src and FAK are vital for optimal invasion [70].

### Penetration into erythrocytes

*Bartonella* species exhibit a complex modus operandi when exploiting erythrocytes, encompassing multiple well-defined stages. The infection odyssey is initiated with the seeding of *Bartonella* into the bloodstream and typically unfolds in a systematic sequence encompassing adhesion, deformation, and invasion, culminating in intraerythrocytic persistence. The binding of *Bartonella* to human erythrocytes is mediated by Trw-T4SS in *Bartonella* lineage 4 (*B. henselae*, *B. quintana* and *B. taylorii*) or flagella of three other lineage species (*B. clarridgeiae*, *B. bovis* and *B. bacilliformis*) to a large extent [50]. The specificity of *Bartonella* in invading erythrocytes seems to be mediated by the supramolecular Trw complex, which includes the surface-exposed T4SS pilus components TrwJ and TrwL [52]. Other studies show that *B. bacilliformis* engages with the α and β subunits of spectrin, band 3 protein, glycophorin A, and both monomeric and dimeric glycophorin B as potential receptors during the initial phases of invading erythrocytes [72]. Post-adhesion and small secretory deformation factors can induce deformation of the erythrocyte membrane, resulting in the formation of pits and grooves on its surface, which provide ideal locations for *Bartonella* to accumulate and gather [73,74]. The ensuing invagination likely ferries the adherent bacteria into the erythrocyte cytosol. This hypothesized pathogen-driven endocytic process is intertwined with the synergistic functions of IalA and IalB [62,75], which are two invasion-associated candidate OMPs in *Bartonella* [60]. Subsequently, *Bartonella* replicates in vacuoles and persists throughout the life of the erythrocytes [76]. Following limited replication, *Bartonella* may be retained within intracellular niches, allowing the infection of blood-sucking arthropods. These erythrocytic sanctuaries offer *Bartonella* an immunological cloak, facilitating their persistence over extended durations [41,75].

Human HPCs, specifically CD34^+^ progenitor cells, can internalize *B. henselae*, acting as a potential niche for pathogens. Therefore, bacterial pathogens can invade and persist in differentiating HPCs, suggesting HPCs as a sanctuary for chronic *Bartonella* infections and recurrent intraerythrocytic bacteraemia [17]. However, erythrocyte progenitor infection *in vitro* may not be the same *in vivo*. Therefore, the reported findings must be further validated *in vivo* in future human studies.

## Induction of vascular endothelial growth factor (VEGF) and host angiogenic responses

Inflammatory response triggered by *Bartonella* infection involves synthesis of diverse cytokines and chemokines [77]. Unlike other pathogenic organisms, *Bartonella* species notably exhibit a pronounced capacity to stimulate the secretion of VEGF, which affects cell growth, migration, and apoptosis by binding to EC receptors [78,79]. As ECs are the primary niche for *Bartonella* infection, this effect may contribute to the persistence of *Bartonella* infection. Consequently, *Bartonella* establishes an implicit infection strategy, rendering it more challenging for the host immune system to eliminate this pathogen.

BadA of *B. henselae* was the first virulence factor found to induce VEGF secretion [63] via a hypoxia-inducible factor-1-dependent mechanism, with its active site located in the head and stalk [28]. Interestingly, Vomps of *B. quintana* share the same VEGF-stimulating activities [68]. VirB/Bep-dependent factors contribute significantly to the angiogenic capacity of *B. henselae*. In certain conditions, such as those observed with *B. bacilliformis*, the absence of VirB/D4-T4SS does not result in the loss of the ability to induce significant angiogenic lesions in infected patients, suggesting that VirB/VirD4-T4SS is dispensable for angiogenesis in at least some species of *Bartonella*. Substantiating this notion is the evidence that the GroEL chaperone of *B. bacilliformis* exhibits mitotic activity in HUVECs, implying its key role in *Bartonella*-induced angiogenesis [81, and 145]. Additionally, BafA, a pro-angiogenic autotransporter identified in both *B. henselae* and *B. quintana*, exhibits affinity for VEGF receptor-2 (VEGFR2), exerting biological effects similar to those of VEGF [82]. A BafA ortholog candidate has also been identified from *B. quintana* (RS02370) and *B. bacilliformis* (RS02470 and RS02475), suggesting that *Bartonella* induces the excessive proliferation of ECs, and subsequent angiogenesis may result from the synergistic effects of multiple virulence factors [83].

The downstream pathway of VEGF stimulation in ECs, including calcium release, and phosphorylation of mitogen-activated protein kinase and phospholipase-Cγ, does not have any specificity [84]. Surprisingly, the VirB/Bep system antagonizes the VEGFR2 signalling cascade. This observation is interesting as VirB/Bep generally acts as a pro-angiogenic factor; therefore, the anti-angiogenic response induced by the VirB/Bep system is presumably an adaptation mechanism for the regulation of potent exogenous angiogenic stimulation to facilitate chronic vascular infection.

## Resistance to lysosomal fusion

Generally, phagocytes play an essential role in killing and degrading pathogens in lysosomes after engulfing. *B. henselae* parasitizes macrophages and ECs for a prolonged time. A significant mechanism underlying this prolonged survival is the ability to subvert lysosomal trafficking, thereby preventing lysosomal degradation. This perspective is primarily derived from the analysis of BCVs after 2-h infection of murine macrophages and human ECs, revealing that BCVs lack typical markers of endocytic pathways and remain unaffected by processes, such as acidification or fusion with lysosomes. Collectively, these observations imply that *B. henselae* occupies a distinct non-endocytic compartment [16]. Evidence also indicates that, upon internalization by macrophages and human ECs, deceased *Bartonella* organisms promptly follow the endosomal route and swiftly merge with lysosomes. In contrast, this process is noticeably delayed in live *B. henselae* [16]. Additionally, paraformaldehyde-treated *B. henselae* retained the ability to direct itself towards the macrophage lysosomes. This observation provides robust evidence that the atypical intracellular trafficking of *B. henselae* is contingent on the presence of viable bacteria. Moreover, Kyme et al. further elucidated that *B. henselae* can inhibit the apoptosis of J774A.1 macrophages and induce the secretion of VEGF, which seems to further enhance the ability of lysosome avoidance, as the induction of VEGF by macrophages or monocytes may contribute to the establishment of a paracrine angiogenic loop towards ECs, leading to cell proliferation and thus facilitating the growth of *Bartonella* [45,85].

## Genetic variation

In their evolutionary trajectory, bacteria have incessantly refined their survival strategies to acclimate to various hostile environments. Horizontal gene transfer (HGT), along with genetic mutations and gene recombination, plays a pivotal role in the adaptive evolution of both prokaryotes and eukaryotes and forms the fundamental essence of bacterial evolutionary processes. Facultative intracellular bacteria, such as *Legionella*, *Francisella*, and *Bartonella*, are capable of thriving in the cytoplasm of their natural hosts, but they also possess the capacity to thrive independently as free-living entities, which is attributed to substantial modifications in the coding genes governing their external surface characteristics [86]. According to previous reports, certain strains of *B. henselae* exhibit significant gene rearrangements and DNA amplification due to genetic variation [87]. The genomes of both *B. henselae* and *B. quintana* consist of numerous short tandem repeats, which are prone to rearrangement and may be related to the phase change of surface-exposed antigens, eventually leading to immune escape [88].

### Gene rearrangement and phase and antigenic variations

Phase and antigenic variations are highly efficient tactics harnessed by microbial pathogens to evade the immune system and can change the expression of some immune-dominant surface proteins during infection by altering amino acid composition or turning protein expression on and off, thus sustaining enduring infections within mammalian hosts. To date, our understanding of the antigenic variation in *Bartonella* is relatively limited. The current conclusions stem primarily from the knowledge of *B. henselae* and *B. quintana*, revealing a trend of genomic rearrangement that may ultimately result in phase variation of surface-exposed antigens [88]. As the reservoir host mounts an antibody-mediated defence against the invading *Bartonella*, robust selective pressure emerges, likely fostering the evolution of clonal populations in which *Bartonella* has either revised its surface-exposed antigens or has altogether ceased its antigenic expression owing to phase variation or complete gene deletion [89]. Intriguingly, the vast majority of these dynamically altered structures, including adhesins, qualify as virulence determinants that are pivotal for pathogen colonization or persistence [90–92]. Additionally, antigenic variation not only confers resistance against host defences but also fine-tunes adhesive interactions, empowering pathogens to adapt and proliferate within ever-changing hostile environments and spread to novel hosts [93].

#### Phase variations in BadA

Adhesion to host cells is an initial stage of the infection cascade that is central to the pathogenicity of *Bartonella*. Extensive *in vitro* studies have elucidated that *Bartonella* species use variably expressed adhesins to bind to diverse host cell types; however, the exact repertoire of these adhesins remains largely undiscovered. BadA is an adhesive protein with high immune capacity; different isolates of *B. henselae* often show genomic and phenotypic differences in BadA. *In vitro* experiments have revealed that the expression of BadA is lost after continuous passages, rendering it incapable of being recognized by the immune system [94]. However, it cannot be completely excluded that such phase variation events of BadA may only be an artefact “illusion” that occurred during the process of laboratory bacterial culture, as once this adhesion ability is lost or weakened, it might be difficult for *B. henselae* to persist in the host cells. Meanwhile, due to the lack of a suitable animal infection model, it is currently impossible to confirm whether the related variation can occur in human infection as well. Riess et al. have emphasized the limited understanding of the BadA mutation mechanism. Under stress, deletion of BadA (phase variation) occurs because of at least two distinct mechanisms: recombination or insertion/deletion of a single base [94]. Structurally, BadA consists of a head domain at the N-terminus, an elongated neck/stalk region with repetitive patterns, and a C-terminal domain that anchors the protein to the membrane. Duplication of repetitive DNA patterns encoding the BadA neck/stalk region may enhance recombination, resulting in a diverse range of surface-bound proteins with distinct attributes [95]. The dynamic interplay of analogous TAA repeats may act as a potential conduit for the swift evolutionary adaptation of immunodominant adhesins [96]. Lending credence to this theory, a recent report revealed that two out of eight strains of examined *B. henselae* lack BadA due to frameshift mutations by identifying a variable genomic badA island [97], suggesting that the highly repetitive neck/stalk region of *badA* potentially enables site-specific recombination or slipped-strand mispairing, leading to reshuffling of *badA*. Consequently, this dynamic alteration in BadA proteins in varying host milieus confers advantages for immune evasion, promoting the colonization capacity of the bacterium.

#### Phase and antigenic variations in OMPs

The genus *Bartonella* is characterized by a diverse repertoire of OMPs, which not only differ across strains but also play pivotal roles in ensuring bacterial structural stability, facilitating host cell adhesion and invasion, and modulating host immune responses. While minor fluctuations in OMP expression have been linked to phase variation, the magnitude of genetic and phenotypic diversity among *Bartonella* phase variants has traditionally been undervalued [33]. A seminal study by Zhang et al. identified a cluster of differentially expressed adhesion proteins in *B. quintana* by examining isolates from an animal model of continuous blood flow [64]. Intriguingly, a clone isolated 70 days post-intradermal inoculation demonstrated a deletion in the genes encoding two of the four Vomps: VompA and VompC [64,98]. The *Vomp* gene family, which encompasses *vompD*, *vompA*, *vompB*, and *vompC*, exhibits a highly conserved tandem arrangement. These genes encode proteins that are localized on the bacterial surface and possess adhesive properties. In particular, VompA and VompC bind to the ECM of host cells, albeit with differential efficacy [64]. Delving further, a molecular analysis of nine primary *B. quintana* human isolates unveiled six distinct restriction fragment length polymorphism profiles using a probe binding to the conserved 5’ region of VompA – C [64]. The absence of one or more *vomp* genes may lead to variable expression of the Vomp family; this is supported by the fact that clinical isolates are often accompanied by the deletion of *vompA* and *vompB*. Persistent fever and recurrent bacteraemia caused by *B. quintana* infection are associated with antigenic variations in Vomps [64].

#### Variations in trw-T4SS

The Trw system, which is essential for erythrocyte colonization, plays a critical role in attachment to host cells [52,99]. Distinct from other identified T4SSs in *Bartonella*, the Trw system exhibits a unique characteristic: the presence of multiple duplicate genes within its gene cluster [88]. An illustrative example is the *trwL* gene, which encodes the pivotal constituent of T-pilus and manifests replication in either seven or eight iterations [88]. Additionally, variability in copy counts across distinct *Bartonella* species has been observed within the genomic segment encompassing the *trwJ*, *trwH*, and *trwI* genes [100]. Studies focusing on homologous genes have proposed that TrwJ plays a pivotal role in facilitating attachment to host cells [101]. In contrast, TrwH and TrwI are responsible for anchoring pili to the outer and inner membranes, respectively [102]. Nystedt et al. revealed that the distinct conservation patterns observed among paralogs of co-amplified *trwJIH* genes in *B. tribocorum* indicate varying selective pressures after gene duplication [100], This suggests that diversifying selection targeting mutations in *trwJ* and/or *trwL* may give rise to various pilus morphologies. The potential for structural variations within the pilus opens new avenues for interactions with diverse surface structures on host cells, thereby encompassing a range of ligands on erythrocyte surfaces. This intricate variability might also contribute significantly to host switching dynamics or act as a broader mechanism, enabling evasion of the immune response by means of antigenic variation.

#### Variations in the bartonella CAMP-Like factor autotransporter (CFA)

CFA, a recently characterized protective antigen, is ubiquitously present across *Bartonella* species in lineage 4, including *B. henselae* and *B. quintana*, and serves as a prime target for neutralizing antibodies. The surface-exposed regions are prone to high variation, extending even to the strain level, suggesting that immune selection pressure in the natural environment could shape the genetic diversity of *Bartonella* and immune escape at the subspecies level of individual strains [103]. This postulation was further bolstered by antigen-antibody binding assays, which revealed pronounced variations in the structural domains of pathogenic *Bartonella* strains isolated from both human and murine hosts. The precise mechanism underlying the variations in CFA remains unclear. Nevertheless, drawing from their substantial similarity to adhesins found in organisms, such as *Plasmodium falciparum*, there is a plausible link between variations in CFA and the presence of the *Bartonella* gene transfer agent (BaGTA). This agent facilitates HGT, thereby enabling the frequent exchange of genetic material among different *Bartonella* strains [104,105]. Such insights suggest that the immunological pressures exerted by antibodies can drive accelerated evolutionary trajectories of protective antigens, giving rise to regions of heightened mutational activity, colloquially termed “mutation hotspots,” which in turn can underpin immune evasion strategies [103].

### HGT by BaGTA

A recent study indicated that *Bartonella* has evolved a sophisticated strategy that preferentially promotes gene exchange within optimal subpopulations. Intriguingly, this mechanism promotes the avoidance of detrimental genetic material exchanges. As a result, it not only safeguards the integrity of the genome, but also expedites the host’s capacity for swift adaptation [104]. Notably, HGT serves as a pivotal conduit for adaptive evolution in both prokaryotes and eukaryotes. Although the ramifications of HGT in bacterial genome dynamics, enhancing adaptability and endurance, are unmistakable, it is imperative to discern that not all gene transfer events have functional implications. Indeed, some occurrences might be mere footprints that reflect a continuum of favourable evolutionary changes [106]. GTAs, now reclassified as viriforms by the International Committee on Taxonomy of Viruses [107], are a key source of HGT among bacterial communities. These GTAs, which are often envisaged as tamed bacteriophages, have evolved molecular apparatuses that facilitate the transfer of bacterial DNA from progenitor cells to closely related recipient cells [108]. Alphaproteobacteria offer compelling evidence of such mechanisms, with entities such as *Rhodobacter capsulatus* harbouring RcGTAs within its genomic architecture, and its homologs are also widely distributed in other bacteria, such as *Paracoccus denitrificans*, *Jannaschia* sp. CSS1, and *Silicibacter pomeroyi* [109]. Another pertinent example is BaGTA, originally identified in *B. henselae*; although characterized by a more stringent host specificity, its presence in *Bartonella* species is incontrovertible [110].

BaGTA serves as a prime example of harnessing elements derived from phages to enable frequent genetic exchanges within bacterial populations. Alongside a multitude of putative proteins, the recognized BaGTA genes encompass components like capsid (bgtG), basal-plate, and tail fibres (bgtC), as well as phage endolysins including endosialidases (bgtD) [104]. The *B. henselae* BaGTA cycling process involves six interconnected steps. (1) GTA is randomly activated within a growing subpopulation of *Bartonella*. (2) BaGTA is encoded upstream of the origin of runoff replication (ROR). (3) *Bartonella* undergoes lysis, leading to the release of GTA, and (4) absorption by receptor organisms. (5) The receptor organisms then take up BaGTA DNA and introduce it into the cytoplasm with the help of various components, such as ComEC, ComM, ComF, and DprA. (6) BaGTA DNA is reintegrated into the receptor organism chromosomes through DprA and host homologous recombination mechanisms [105]. Although BaGTA does not seem to be a random process, it specifically promotes genetic exchange between the most suitable bacterial subsets. Due to the influence of the stringent response signal known as guanosine tetraphosphate (ppGpp), the activation of BaGTA is constrained to rapidly proliferating bacteria [104]. Sustaining the presence of BaGTA in subsequent phases can be attributed to selective pressures that enhance the potential for genetic interchange. This, in turn, accelerates the organism’s ability to swiftly adapt to host defence mechanisms during the course of infection [111]. Although BaGTA may not be directly related to pathogenicity, its role in the adaptive strategies of *Bartonella* within hosts cannot be underestimated. It is plausible that the BaGTA-driven modulation of structural proteins, as exemplified by CFA, is instrumental in immune subversion strategies [111]. This intricate molecular interplay between BaGTA-mediated CFA variations and subsequent ramifications of the pathogenic profile of *Bartonella* warrant further investigations.

## Inhibition of apoptosis and maintenance of the integrity of host cells

### Inhibition of EC apoptosis

Several bacteria elevate the intracellular cAMP concentrations upon infection via modulation of adenylyl cyclase [112] or ADP ribosylation activity [113,114], implicating this modulation as a hallmark of their pathogenicity. *B. henselae* and *B. quintana* disrupt the intracellular cAMP dynamics to counteract apoptosis in mammalian vascular ECs [79], potentially enabling evasion of both antibody-mediated and complement-dependent immune responses [115].

*B. henselae*, the most extensively investigated *Bartonella* species, elevates the intracellular cAMP levels to antagonize cytotoxic T lymphocyte-dependent cell death and maintain the integrity of ECs via VirB/D4-T4SS and BepA [116–118]. Although the precise underlying mechanism remains unclear, prevailing data indicate that all *B. henselae* Beps harbour a conserved region within their C-terminus, termed the Bep intracellular delivery (BID) domain [118], which is linked to the upregulation of cAMP and promotion of the expression of its responsive genes. Supporting this notion, pharmacological inhibition of cAMP degradation emulates a similar anti-apoptotic phenotype [119], underscoring the centrality of cAMP in the anti-apoptotic function attributed to BepA. However, elucidating the exact molecular nexus through which BepA of *B. henselae* mediates cAMP surge remains a challenging endeavour. As BepA localizes in the plasma membrane after secretion into the cytoplasm, it may directly interact with the host cell AC, thereby potentiating Gαs (α subunit of the G protein)-driven cAMP biosynthesis [120]. The anti-apoptotic mechanisms instigated by *Bartonella* not only fortify the intracellular niche, aiding in completion of its developmental cycle within the host cells, but also furnish a refuge, effectively shielding it from both antibiotic intervention and immune attacks.

### Alleviation of the harmful effects on host cells

Beps are virulence factors necessary for colonization in mammalian hosts. Intriguingly, their intrinsic interactions can inadvertently perturb host cellular signalling cascades, potentially culminating in deleterious consequences. Therefore, certain *Bartonella* species have evolved a repertoire of strategies to ensure the preservation of host cellular integrity, which is a prerequisite for their sustained intracellular existence. A notable example is *B. henselae* BepE, which has demonstrated its capacity to safeguard ECs against fragmentation and counteract the impairment of DCs migration caused by BepC and other Beps. This action permits *B. henselae* to endure and thrive within its host, thereby facilitating its subsequent dissemination.

Empirical evidence from *in vitro* experiments involving HUVECs that when infected with a BepE-deficient *B. henselae* strain (ΔbepE), the cells manifest a fragmentation phenotype. This cellular aberration stems from the Bep-mediated disruption of rear-edge detachment during migratory processes [121], suggesting that BepE has a protective effect on cell integrity upon *B. henselae* infection [121]. In stark contrast, several other Bep isoforms (namely, BepC, A, B, and G) appeared to compromise cellular migration, subsequently leading to fragmentation. This juxtaposition implies an antagonistic functional dynamic between BepE and Bep isoforms, although the intricacies of these interactions remain unclear. The observed cellular fragmentation attributed to Bep is presumably linked to the suppression of small GTPases, particularly RhoA. This hypothesis is supported by the substantial reduction in this effect upon prior application of the Rho inhibitor I [121]. Delving deeper, it has been revealed that in the presence of the ROCK inhibitor, Y27632, BepE demonstrated an inability to enhance stress fibres. This observation implies that BepE may function as a potential factor that directly or indirectly triggers RhoA activation. Then phosphorylate downstream ROCK, thereby inducing cytoskeletal reorganization, cell migration, and stress fibre formation, ultimately affecting endothelial permeability, tissue constriction, and growth. Although the precise interplay and localization of BepE within the RhoA/ROCK signalling axis remain to be elucidated, recent reports indicate that BepE in *B. henselae* can modulate the RhoA pathway via its BID domain, thereby alleviating the deleterious effects of BepC [122].

### Modulation of the IL-8/Anti-apoptotic pathway

*B. henselae* employs intricate mechanisms to stimulate angiogenesis and cellular proliferation, involving the activation of nuclear factor (NF)-κB-dependent inflammatory gene expression [117,123,124]. Notably, within infected HUVECs, *B. henselae* induces the synthesis of IL-8 [85,117], which is important in regulating the Bcl-2/Bax ratio [125]. Furthermore, the inhibitory effects of *B. henselae* has been shown to effectively suppress EC death, similar to the caspase inhibitor, Z-VAD-FMK [79]. These observations suggest that *Bartonella*-triggered IL-8 May orchestrate angiogenesis by modulating the anti-apoptotic pathway within ECs, thus ensuring the stable growth of the primary niche.

## Avoiding TLR recognition

During intracellular habitation, the invading Bartonella inevitably confronts the threat posed by the host immune system. Consequently, downregulation of the immune response is imperative for successful parasitism. For instance, *Bartonella* can modify their surface structures, such as LPS and flagella, to attenuate TLR signalling. Beyond these modifications, *Bartonella* orchestrates more intricate manipulation by disrupting the delicate equilibrium between pro-inflammatory and anti-inflammatory cytokines. Moreover, they possess the remarkable ability to counteract the lytic effects of host lysozymes. Although current scientific inquiries present more conundrums than resolutions, these adept adaptations augment the odds of *Bartonella*’s sustained survival in the host.

### Inefficient activation of the TLR4 pathway by LPS

LPS is an integral component of the outer membrane of gram-negative bacteria. It encompasses distinct domains, including lipid A as the lipid component, a central core polysaccharide, and an external polysaccharide segment recognized as an O-polysaccharide. Lipid A is instrumental in endotoxic activity and is identified by TLR4 as a pathogen-associated molecular pattern [126]. Even at minimal concentrations, the lipid A component of LPS engages with a membrane receptor complex composed of TLR4, MD-2, and CD14. This interaction initiates the innate and adaptive immune cascades to eradicate invading pathogens. However, the structure of lipid A varies considerably among bacterial species, particularly in terms of the number of fatty acid moieties, chain length, and the presence of terminal phosphate residues that can undergo modifications. Such variations may enhance resistance to host cationic antimicrobial peptides or alter their interaction with the TLR4 complex, affecting its recognition [127], enabling bacteria to develop strategies to evade detection by the innate immune system. For instance, in *Helicobacter pylori*, *Escherichia coli*, and *Pseudomonas aeruginosa*, the lipid A has been associated with suboptimal activation of the TLR4 signalling pathway [127].

Interestingly, *B. henselae* 49882T exhibited an intriguing feature in which its lipid A O-chain composition was distinct. This unique configuration is characterized by the bisphosphate backbone of a 2,3-diamino-2,3-dideoxy-glucose disaccharide embellished with an atypical assembly of long fatty acids, specifically pentacylation [128]. Subsequent experimental analyses suggested that these distinct structural elements may underlie their diminished endotoxic potency and hinder the induction of a robust immune response. To some extent, LPS from *B. quintana* can counteract TLR4-mediated cytokine transcription in monocytes when stimulated by *E. coli* LPS [129], indicating that such specific variants may fail to sufficiently engage TLR4 signalling pathways to elicit an effective immune response [129]. Mechanistically, *B. quintana* does not function as a typical endotoxin in structure as that in *E. coli*, but as a lipooligacharide with five fatty acid tails (two of C12, two of C16, and one very long C26), and the aggregate structure is different from that of the typical LPS, which hides the binding epitopes in LPS to the TLR4 receptor necessary for cell signalling [130]. Although *Bartonella* LPS has limited ability to activate TLR4, its precise role and interaction dynamics with the host immune system, especially considering the prolonged residency of *Bartonella* within the host, remain elusive. Additionally, no similar LPS have been reported in other *Bartonella* species.

### Evasion of the TLR5-mediated immune recognition by Flagella

Flagella are motile filamentous helical protein appendages that emanate from the surface of certain bacteria. In the genus *Bartonella*, only *B. schoenbuchensis* [131], *B. bacilliformis* [59] and *B. clarridgeiae* [58,132] possess flagella and polymerize into rod-like structures, which serve as dynamic factors of bacterial movement. Generally, the flagellum acts as a ligand for TLR5, initiating a proinflammatory response against invading microorganisms [133]. However, not all flagella of bacteria share the same characteristics, as *B. bacilliformis* fails to trigger TLR5-dependent activation of NF-κB [134]. Although the flagellum mutant retains its motor ability, evasion of a TLR5-dependent immune response is beneficial for the sustained survival of *Bartonella*. However, the mechanisms underlying this evasion remain unclear. The TLR5 stimulatory region is nestled within its N-terminal D1 domain, predominantly focusing on amino acid residues 89–96 [134]. Additionally, the activation of TLR5 requires contributions from both the D2–D3 and C-terminal D1 domains. Therefore, it is plausible that the flagellum of *B. bacilliformis* possesses a structural uniqueness that either diminishes its binding affinity for TLR5 or predisposes it to interact with alternate TLR5 domains. Comprehensive studies are required to determine the exact nuances governing this interaction.

## Disruption in the balance of pro-/Anti-inflammatory cytokines

Certain chronic bacterial pathogens skilfully evade eradication of innate immune system, orchestrating a prompt transition from a pro-inflammatory phase to a tissue-reparative, anti-inflammatory state [135]. Regulatory protein signal transducer and activator of transcription 3 (STAT3) acts as a nuclear transcription factor regulating the genes involved in the cell cycle, cell survival, and immune responses. It plays a constructive role in maintaining the equilibrium between proliferation and apoptosis, while also contributing to self-antigen tolerance and antigen presentation [136]. However, this crucial regulatory system can be co-opted, as exemplified by specific viral and bacterial pathogens that capitalize on the activity of STAT3 to their advantage [135]. Recent investigations have revealed a distinctive ability of the *B. henselae* effector BepD: its capacity to activate the canonical STAT3 pathway. This activation leads to the restraint of pro-inflammatory cytokine tumour necrosis factor-α secretion and the enhancement of IL-10 production [26]. Mechanistically, BepD uses various phosphorylated EPIYA-related motifs to trigger the activation of STAT3 in immune cells via an intrinsic pathway depending on the tyrosine kinase, c-Abl. This orchestrated activation triggers a phenotypic shift from a proinflammatory to an anti-inflammatory response. Consequently, the scaffold of BepD phosphorylated on tyrosine residues takes centre stage as a pivotal signalling hub for the intrinsic activation of STAT3. Importantly, this activation operates autonomously via the conventional STAT3 activation pathway, involving transmembrane receptor-associated Janus kinases [26].

## Candidate factors: ingestion and detoxification of exogenous heme

Haem, a ubiquitous molecule predominantly bound to haemoglobin in the mammalian bloodstream, is a paramount source of iron for the host [137]. Virtually every bacterial pathogen requires haem for successful vertebrate infection. However, this molecule presents a conundrum; excessive haem concentrations are cytotoxic owing to its potent redox properties. The iron within haem readily generates harmful reactive oxygen species, leading to lipid peroxidation, DNA damage, and other detrimental effects on cells [138]. Notably, *Bartonella* experiences varying haem concentrations [56] within two distinct habitats: mammalian hosts and the gut of the obligate haematophagous arthropod vector. To navigate these dynamic environments, *Bartonella* species must possess sophisticated haem regulation and scavenging mechanisms to satisfy their nutritional requirements and ensure self-preservation. Remarkably, *B. quintana* exhibited the highest in vitro haem requirement. Nevertheless, genomic analyses have confirmed that neither *B. quintana* nor *B. henselae* possesses the capacity for de novo haem synthesis. Notably, homologs encoding porphyrin biosynthetic enzymes are absent in these species [88], indicating that *Bartonella* obtains haem from the surrounding environment to meet its nutritional needs.

A quintet of haem-binding proteins (Hbps), HbpA – E, has been delineated [80,139]. Interestingly, the unique utilization of its array of Hbps in a unique fashion has been postulated in *B. quintana*. HbpA, HbpD, and HbpE are utilized when growth is constrained by haem, as observed in the circulation of reservoir hosts. Conversely, HbpB and particularly HbpC come into play when encountering exceedingly high and toxic haem concentrations, such as those encountered within the midgut lumen of its arthropod host, the louse [139]. Although the presence of haem-binding receptors in *Bartonella* remains uncertain, Roden et al. postulated that HbpC might directly bind to haem through an innovative mechanism, or make the outer membrane more permeable to haem, thereby facilitating its passage. Notably, HpbC localization has been confirmed in the outer membranes and outer membrane vesicles (OMVs) of *B. henselae* [55]. Additionally, *hbpC* transcription exhibits sensitivity to temperature, evident in increased *hbpC* expression at 28 ℃ compared to 37 ℃. This corresponds with an elevated production and release of haem-containing OMVs at 28 ℃, which serves to safeguard *B. henselae* from the hazardous levels of environmental haem within the arthropod gut [55]. Thus, regulation of HbpC expression has emerged as a strategic approach for *Bartonella* to acquire or detoxify exogenous haem. It has also emerged as a potential determinant survival of *Bartonella* and evasion from the immune system.

### Binding to fn and evasion of immune recognition

Fn, which encompasses both plasma Fn in bodily fluids and cellular Fn on cell surfaces, plays a pivotal role as a component of the ECM that connects cells with their surrounding environment and other ECM constituents, such as collagen and laminin [140]. The interaction between *B. henselae* and ECM components, such as Fn, is of significant importance in bacterial adhesion and invasion of host cells. Studies have also proposed that the neck/stalk domain of BadA can bind to plasma Fn, enabling pathogens to cloak themselves with plasma Fn while retaining the ability to adhere to host cells through pericellular Fn. This binding to soluble Fn may facilitate the dissemination of *B. henselae* to secondary sites, whereas *Bartonella* coated with Fn could aid in evading host immune surveillance [141,146].

## Conclusion and future perspectives

Advancements in bacterial genomics and development of animal and *in vitro* cell infection models have enhanced our understanding of the molecular and cellular basis of *Bartonella* infections. Notably, *B. henselae*, *B. quintana*, and *B. bacilliformis* infections in their reservoir mammalian hosts predominantly manifest as persistent intraerythrocytic bacteraemia. Although this trait is consistent, the clinical manifestations vary, ranging from asymptomatic states to subclinical presentations and minimal morbidity to pronounced mortality, occasionally escalating to life-threatening cases across species. Enduring intraerythrocytic bacteraemia hints at the unique adaptation of this species to their respective hosts, complemented by the deft evasion of host immune responses. In this review, we introduced the main strategies of *Bartonella* to evade host immune system clearance. Occurrence of symptoms depends on various factors, including the virulence of *Bartonella*, host immune status, and cytokines in the immune system. The major challenge in investigating *Bartonella* is the establishment of a suitable animal model that closely mimics the human infections. This limitation hampers experimental design and data analysis, hindering the comprehensive assessment of *Bartonella* immune evasion mechanisms. Therefore, establishment of animal models that better reflect human infections is necessary to explore the mechanisms of *Bartonella* infection and immune evasion and assess the efficacy of novel therapeutic strategies and vaccines. Furthermore, *Bartonella* affects various host, including mammals, birds, and reptiles. By the way, *B. bacilliformis* is quite different in terms of clinical disease manifestations and the absence of the VirB/D4-T4SS. This diversity complicates the study of immune evasion by *Bartonella* as different hosts may elicit different immune responses to these species. Moreover, diverse *Bartonella* strains induce distinct immune reactions [142]. Therefore, it is crucial to investigate the variations in host immune responses, including immune response patterns, changes in cell types, and alterations in immune factors, to clearly understand the manner in which these differences affect immune evasion and severity of infection. This is an important direction for future research.

Despite the various mechanisms employed by *Bartonella* to evade host immune system attacks, such as altering the structure of surface antigens and manipulating immune cell functions, our comprehensive understanding of these mechanisms remains limited. *Bartonella* species survive and replicate within the host, leading to long-term persistent infections and pathological changes. Therefore, further studies are necessary to explore the interactions between *Bartonella* and the host immune system, specifically the mechanisms of surface antigenic variation, immune evasion strategies, and molecular mechanisms of bacterial survival in the host cells. With the rise of modern technologies and methods, our research on *Bartonella* is becoming more optimistic. Various techniques providing detailed insights, such as single-cell sequencing, high-throughput proteomics, and functional genomics, have been developed. By combining datasets from diverse sources and creating robust mathematical models, we can shed light on the overarching frameworks and temporal trajectories of immune evasion, thereby revealing the intricate relationship between microbial infection and host immune response. This will also help to decipher the complex interactions between *Bartonella* infection and host immune evasion. Therefore, future studies need to adopt a collaborative ethos that cuts across disciplinary silos, including immunology, microbiology, and bioinformatics. Such efforts will not only facilitate data dissemination and support open science but also accelerate scientific advancements, leading to groundbreaking and transformative discoveries.

In conclusion, future research on *Bartonella* immune evasion should focus on elucidating the mechanisms of immune escape, differences among various host immune responses, and mechanisms of persistent infection. By integrating systems biology approaches and fostering interdisciplinary collaboration and data sharing, future studies can enhance our understanding of *Bartonella* infection and immune evasion mechanisms, facilitating the development of novel approaches for its prevention and treatment.

## Data Availability

No data were generated or analysed in this work.
